# Antioxidant Properties of Gluten-Free Pasta Enriched with Vegetable By-Products

**DOI:** 10.3390/molecules27248993

**Published:** 2022-12-16

**Authors:** Amel Betrouche, Lorenzo Estivi, Davide Colombo, Gabriella Pasini, Leila Benatallah, Andrea Brandolini, Alyssa Hidalgo

**Affiliations:** 1 Food Engineering Laboratory, Institute of Nutrition, Food and Agri-Food Technologies (GéniAAl-INATAA), University Frères Mentouri Constantine 1 (UFMC1), 325 Route de Ain El Bey, Constantine 25017, Algeria; 2 Department of Food, Environmental and Nutritional Sciences (DeFENS), University of Milan, Via Celoria 2, 20133 Milan, Italy; 3 Department of Agronomy, Food, Natural Resources, Animals and Environment (DAFNAE), University of Padua, Viale dell’ Università 16, 35020 Legnaro, Italy; 4 Research Centre for Animal Production and Aquaculture (CREA-ZA), Council for Agricultural Research and Economics, Via Piacenza 29, 26900 Lodi, Italy

**Keywords:** carotenoids, fava bean, flavonoids, linseed meal, phenolic acids, rice, tocols, tomato pomace, waste

## Abstract

The only therapy for coeliac disease patients is to completely avoid foods containing gluten, a protein complex common in several small-grain cereals. However, many alternative gluten-free foods available on the market present nutritional deficiencies. Therefore, the aim of this research was to evaluate the composition and the antioxidant properties of gluten-free pasta enriched with 10% or 15% of tomato waste or linseed meal, two food industry by-products. The traits analysed were protein, lipid, ash and fibre content, heat damage, tocols, carotenoids and phenolics composition (by HPLC), antioxidant capacity, and pasta fracturability. The enriched pastas contained more fibre and lipids than the control, while the protein and ash values were similar. The addition of tomato and linseed waste improved tocols concentration but had no effect on carotenoids content. The free soluble polyphenols increase was similar for both by-products and proportional to the enrichment percentage, while the bound insoluble polyphenols were higher in linseed-enriched pastas. The samples with linseed meal showed the greatest antioxidant capacity and, at 10% addition, the highest fracturability value. In conclusion, the addition of tomato and linseed by-products significantly increases the presence of bioactive compounds (particularly polyphenols), improving the nutritional value of gluten-free pasta.

## 1. Introduction

Coeliac Disease (CD) is a chronic small intestinal immune-mediated enteropathy triggered by exposure to dietary gluten in genetically predisposed individuals [1]. A meta-analysis [2] recorded that the worldwide prevalence of celiac disease is 1.4% based on blood tests and 0.7% based on biopsy. Additionally, they observed that its prevalence ranged from 0.4% in South America to 0.5% in Africa and North America, to 0.6% in Asia, reaching to 0.8% in Europe and Oceania. A strict and life-long gluten-free (GF) diet is the only available therapeutic treatment for people with CD. Only foods made from naturally GF ingredients and that contain no more than 20 parts per million gluten (i.e., the lowest level that can be reliably detected by scientific analytical tools) can be defined as GF products [3,4,5]. Currently, a broad variety of GF products (e.g., bread, cakes, muffins, pasta/noodles) is available to coeliacs. However, many GF foods are poor in minerals and bioactive components, and consequently are nutritionally inferior to the wheat-based foods they replace. Therefore, more attention and research are needed to improve the nutritional aspects of these products [6].

Pasta is appreciated and consumed all over the world for its taste, ease of preparation, low cost, and long shelf life. Good-quality pasta is prepared exclusively from durum wheat, but other types (e.g., noodles, egg pasta, etc.) are made from bread wheat or allied species. However, all these pastas are not suitable for people with CD, pushing the industry to look for and manufacture products whose main ingredients are flour from gluten-free species (rice, corn, sorghum, amaranth, quinoa, buckwheat, fava bean, pea, chickpea, etc.), and/or starch from corn, rice, and potato, with the addition of protein, gums, and emulsifiers which may partially act as gluten substitutes [7,8]. Rice is very suitable for GF foods manufacturing because of its high starch content, neutral taste, white colour, high digestibility, and hypoallergenic properties [9], while legume flours, rich in high-quality proteins, fibres, starch, and minerals, represent a good alternative and/or addition [10].

These last years have seen a sharp increase in GF pasta demand, which is consumed not only by a growing number of coeliacs but is also actively sought-after by non-coeliac people who wish to exclude gluten-based products from their diet for perceived health reasons. GF pasta is usually prepared in two ways. The first approach focuses on the use of heat pre-treated GF flours, containing mostly gelatinized starch; then, the pre-treated flour can be shaped into pasta by the continuous extrusion press commonly used for durum wheat semolina pasta making. The second approach (extrusion cooking process) treats native flour with steam and extrudes it at a high temperature (>100 °C), promoting starch gelatinization directly inside the extruder–cooker. The conventional extrusion induces the creation of small crystalline regions, resulting in low water absorption, while the extrusion cooking process leads to strong interactions between amylopectin and/or amylose molecules, thus giving a product with low cooking loss and high firmness [7].

The industrial processing of fruits and vegetables generates large by-product waste in the form of peels, cores, pomace, unripe and/or damaged fruits and vegetables [11], whose disposal may be expensive and can have a negative environmental impact. Therefore, reducing food waste is a priority to move towards more sustainable food systems. Traditionally, fruit and vegetable by-products are reutilized as animal feed, processed into biogas, or composted to obtain biofertilizer [12]. A worthier approach, leading to both economic and social benefits, may be the transformation of waste into functional foods ingredients [13,14], because these by-products are often a rich source of nutrients and other functional compounds. For example, tomato peels and seeds are rich in fibres [15] and proteins [15,16]. Additionally, they contain antioxidant compounds like polyphenols [15,17], tocopherols [17], and carotenoids (e.g., β-carotene [15] and lycopene [15,18]), which exert positive health effects against oxidative stress due to reactive oxygen species (ROS) at the cellular and DNA levels, contributing to preventing the insurgence of age-related diseases and cancers [19,20,21]. Similarly, the linseed cake, a by-product of linseed oil extraction, has abundant protein water-soluble fibre and lignan concentrations [22] along with high phenolic acids content [23].

Some by-products have been successfully added to traditional products, improving their nutritional value but often altering their technological quality. For example, pasta enriched with 15% tomato peels has higher levels of carotenoids and dietary fibre than the control pasta, but possesses lower sensory scores for elasticity, odour, and firmness; nevertheless, the tomato peel hydrocolloids enhance adhesiveness and bulkiness, without compromising other physical and chemical properties [24]. Linseed meal was used as an additive in bread [25]. The addition of 15% linseed flour and linseed marc gave breads higher nutritional value, increasing protein content, fat, and mineral components by 11.06, 4.03 and 1.84 g/100 g, respectively, while maintaining acceptable sensory properties [26]. The addition of food industry by-products to gluten-free foods might be even more appealing [27], because of the above-mentioned nutritional shortcomings. For example, in gluten-free pasta the addition to rice flour of a potato peel water extract—*Psyllium* husks gel (50–50 ratio)—increased the mineral content, total phenolic content, and antioxidant activity without affecting the mechanical properties [28]. Similarly, gluten-free noodles prepared with resistant rice starch, defatted rice bran (5%), and xanthan gum (2.5%) exhibited good protein, fibre, and ash content coupled to low glycaemic index and high sensory acceptability [29].

The addition of vegetable wastes to improve the nutritional properties of gluten-free products is still in its infancy, and very few studies are available. Therefore, the aim of this research was to evaluate the composition and the antioxidant properties of gluten-free pasta enriched with different quantities of two food industry by-products: tomato waste and linseed meal.

## 2. Results and Discussion

### 2.1. Flours

#### 2.1.1. Chemical Characteristics

Table 1 reports the protein, ash, lipid, and furosine content of the raw materials used in the production of gluten-free pasta; the LSD test results are also presented. Fava bean flour and linseed meal had a protein content about twice that of the tomato waste and about four times that of the rice flour, while the highest ash and lipids concentrations were in linseed meal, followed by TP, bean flour, and rice. The results confirmed that all these three groups of compounds are scarce in rice flour, and hence the nutritional value of rice is mainly due to carbohydrates. Furosine, a compound which develops from the acid hydrolysis of the first stable products of the Maillard reaction (the Amadori compounds), was found in small and decreasing quantities in TP, bean flour, and linseed meal (probably due to drying treatments) but was not detected in rice flour.

The composition of the four raw materials was comparable to the data reported in the literature. In particular, rice flour values were consistent with the protein (7.63–9.62 g/100 g DM), ash (0.63–1.32 g/100 g DM), and lipid (0.68–1.64 g/100 g DM) contents described by Devraj et al. [30], Goufo et al. [31], and Culetu et al. [32]. Similarly, fava bean flour values were within the ranges (22.39–31.07, 2.95–4.46, and 0.99–2.20 g/100 g DM for protein, ash, and lipid, respectively) reported by De Angelis et al. [33] for 13 cultivars. On the other hand, the TP results were inferior to those (15.04–23.07, 3.08–7.01, and 5.04–20.05 g/100 g DM for protein, ash, and lipid, respectively) described by several authors [34,35,36]. However, the by-products from tomato processing have a very heterogeneous composition depending on the relative abundance of seeds and peels, which contain different levels of proteins (25.50 and 14.47 g/100 g DM) and lipids (17.15 vs. 1.77 g/100 g DM) [36]. Finally, the linseed extraction cake composition was comparable with the Kaur et al. [37] samples, which had 28.79, 6.12, and 18.69 g/100 g DM of protein, ash, and lipids, respectively, while Mannucci et al. [38] found higher protein (30.40 g/100 g DM) and lower lipids (9.81 g/100 g DM) content.

#### 2.1.2. Lipophilic Antioxidants

Total carotenoids (mainly lycopene + β-carotene) were plentiful in TP, followed by fava bean flour (>90% lutein) and linseed meal (about 70% lutein), while rice flour was almost devoid (Table 2). The total carotenoid levels of TP were an average value between those reported in peels (15.3 mg/kg DM, expressed as lycopene) and seeds (2.6 mg/kg DM) [39], but were significantly lower than the lycopene contents (98.2–690.9 mg/kg DM) observed by some authors [40,41,42]. In linseed meal, carotenoids concentrations (5.17 mg/kg DM, mostly lutein) were about double those found in our sample [38], while in fava bean they were lower than ours (1.38–2.27 mg/kg dry legumes) [43]. In general, a comparison between different experiments may be difficult and sometimes misleading, because different varieties, environments, techniques, temperatures, and storage conditions strongly influence the outcome [44].

The raw material with the highest tocols content, mostly β-tocotrienol (about 68%) and γ-tocopherol (about 31%), was linseed meal. A good concentration was also found in fava bean flour and in TP, where the two main components were γ-tocopherol (90% and 78%, respectively) and α-tocopherol (6% and 20%, respectively). Once again, rice flour showed the lowest values. In general, the results reported in the literature are inferior to ours. For example, linseed cake contained 25.63 mg/kg DM total tocols, including γ- (93%), α- (4%) and δ-tocopherol (3%) [38], while the fava bean flour contained 8.99–13.34 mg/kg DM total tocols, with a marked prevalence (85–87%) of the γ homologue [43,45]. However, in white rice significant quantities of tocopherols (0.21–10.90 mg/kg DM) and tocotrienols (3.85–24.49 mg/kg DM) were detected [46].

#### 2.1.3. Hydrophilic Antioxidants

The free soluble polyphenol composition of linseed meal is unavailable due to interferences in the chromatogram, probably because its abundant lipids masked most of the peaks and prevented their identification. The fava bean and tomato by-product had the greatest content of total free polyphenols (Table 3). In fava beans they were mainly catechin (58%) and protocatechuic acid (27%), followed by syringic acid (6%), tyrosol (4%), and epicatechin (4%), while in tomato by-product they were tyrosol (44%), rutin (13%), naringenin (12%), and syringic acid (10%), as well as other compounds in lesser quantities, such as quercetin derivative (7%), epicatechin (5%), protocatechuic acid (4%), and other phenolic acids. No appreciable quantities of free soluble polyphenols were found in rice.

In the flours of ten Australian fava bean varieties, 279.7–402.5 mg/kg of free polyphenols were found [47]; some varieties also had a content of syringic acid (72.4–122.5 mg/kg) like ours, while protocatechuic acid was scarce (1.29–2.93 mg/kg) and catechin was more abundant (191.0–297.0 mg/kg). Similarly, a prevalence of catechin (455.6 mg/kg DM), followed by epicatechin (23.6 mg/kg DM) and syringic acid (14.3 mg/kg DM) was reported [48]. We did not detect anthocyanidins, found in small amounts (3.74–9.25 mg/kg) by Kan et al. [43]. The tomato waste analysed by Szabo et al. [42] was generally richer in free phenolic compounds (1018–4077 mg/kg DM), particularly rutin, naringenin chalcone, quercetin-triglucoside, and 3–4-dicaffeylquinic acid; a superior content (2861 mg/kg DM), with a prevalence of naringenin and naringenin chalcone, was reported by Abbasi-Parizad et al. [49]. However, lower concentrations (90 mg/kg defatted DM) are also reported [50]. Being a mixture of peels and seeds, tomato by-products composition varies according to their ratio, because the seeds are richer in phenolic acids and poorer in flavonoids [51]. These differences may be a consequence of the diverse drying conditions or treatments performed on the pomace. The linseed extraction cake studied by Kaur et al. [37] showed a content of 246 mg/kg DM free phenols (34% phenolic acids, 64% flavonoids, and about 1.6% resveratrol), while in rice flour they were almost negligible: 2.8 mg/kg DM [52].

The bound Insoluble polyphenols (Table 3) were maximum in the TP, particularly rich in the flavonoids naringenin (56%), quercetin (19%), and epicatechin (12%), but also in the phenolic acids cinnamic derivative (4%), ferulic (3%), and 4-hydroxybenzoic (2%). Perea-Domínguez et al. [50] found a very similar result for total bound phenolics (1043 mg/kg defatted DM), confirming the predominance of naringenin and quercetin (723 and 169 mg/kg DM); in addition, the authors observed a good concentration (404 mg/kg DM) of a fraction, the soluble conjugated phenolics, which we did not study. Linseed meal also contained a sizeable bound polyphenols content, with a strong contribution of protocatechuic acid (49% of total bound polyphenols) and ferulic acid (40%). A far superior value (5684 mg/kg DM) was reported [37]. Modest bound polyphenols quantities (57.7 mg/kg DM), mainly represented by ferulic acid (89%), were found in rice flour [48]. Finally, the bound polyphenols in fava bean were scarce, and largely inferior to the 73.92 mg/kg DM described in the literature [53].

#### 2.1.4. Antioxidant Capacity

Analysing the antioxidant capacity of the hydro-soluble compounds, the fava bean flour, which had the highest total free polyphenols concentration, consistently had the greatest ABTS-MeOH value (107.69 mmol TE/kg), followed by linseed meal (60.73 mmol TE/kg) and TP (15.54 mmol TE/kg); modest or undetectable values were recorded for rice flour. When the FRAP method was used, the fava bean flour methanolic extract scored 18.14 mmol TE/kg, lower than that linseed meal (36.18 mmol TE/kg) and slightly higher than tomato pomace (15.54 mmol TE/kg). The ABTS values of the liposoluble compounds—extracted with hexane—were modest in all raw materials, indicating that this fraction was less relevant for antioxidant capacity.

### 2.2. Pasta

#### 2.2.1. Chemical Characteristics

**Figure 1:**
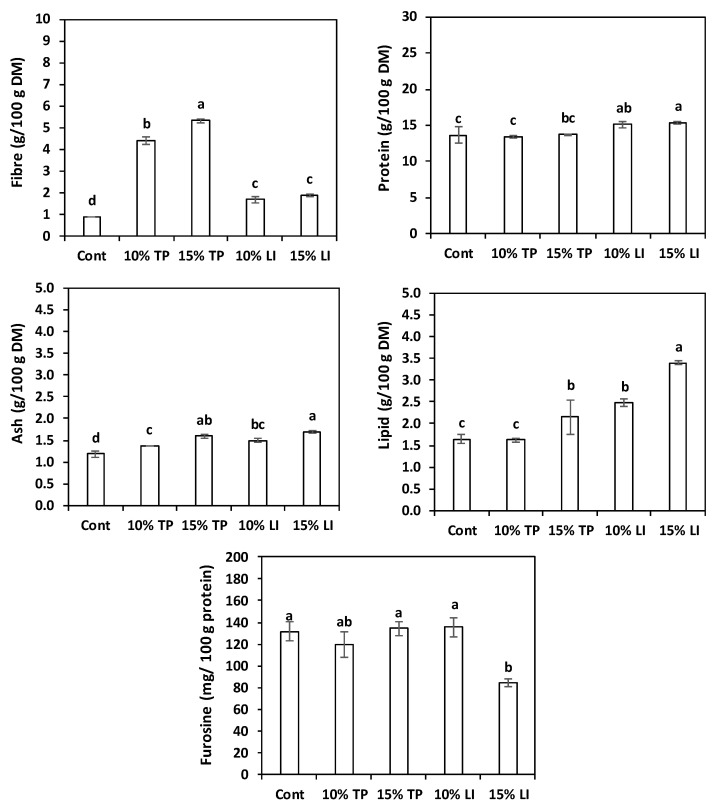
Chemical characteristics and heat damage of dried pasta prepared with rice flour and fava bean flour (Cont) or enriched with different percentages of tomato pomace (TP) or linseed meal (LI). The error bars represent the standard deviation; different letters indicate significant difference (*p* < 0.05) between the samples.

Figure 1 depicts the centesimal composition (fibre, protein, ash, lipid) and the furosine content of the five pasta types tested. By-products enrichment increased the fibre content of the samples, especially when the TP was employed; the highest value was reached with 15% TP. This is consistent with Padalino et al. [24], who enriched durum wheat pasta with the very same percentages of tomato pomace, finding, however, much higher levels of dietary fibre (16.80 and 19.75 g/100 g) due to the higher content in their flour. The protein content of the control and of the TP-enriched pastas was similar, probably because the protein concentration of the basic formulation was already high, while the linseed meal enrichment provided slightly higher values. On the contrary, dilution of protein in TP-enriched pastas was previously reported [24]. Our protein values in linseed-cake-enriched pastas (15.38–15.89 g/100 g DM) were consistent with those found by Zarzycki et al. [54] in semolina pastas enriched with 9–17% linseed oil cake (14.93–16.51 g/100 g DM).

Ash and lipid increased with augmenting enrichment levels. The substitution effect was particularly evident in the pasta with linseed meal, because of the residual lipid content. Zarzycki et al. [54], in the above-mentioned study, found similar values of ash (1.39–1.73 g/100 g), but lower fats (0.8–1.8 g/100g) because of the inferior lipid content of their cake (12.41 g/100 g).

On the other hand, furosine was always low; one investigation, involving more than 100 durum wheat pastas, reported a range of 226–506 mg/100 g of protein [55]. Moreover, the extremely small values found in pasta enriched with 15% linseed meal possibly hint to a possible role of lipids in thermal damage prevention.

#### 2.2.2. Lipophilic Antioxidants

**Figure 2:**
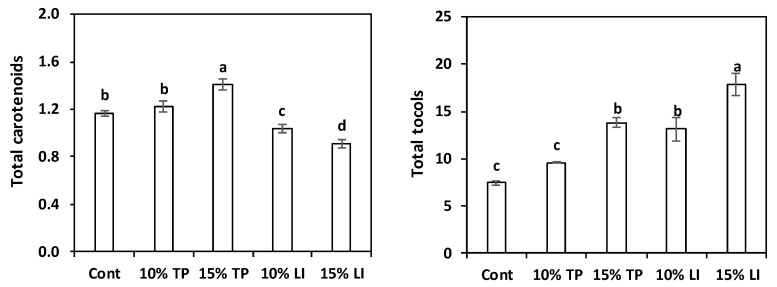
Total carotenoids and total tocols content (mean ± standard deviation; mg/kg DM) of pasta prepared with rice flour and fava bean flour (Cont) or enriched with different percentages of tomato pomace (TP) or linseed meal (LI). The error bars represent the standard deviation; different letters indicate significant difference (*p* < 0.05) between the samples.

The total carotenoid content of the enriched pastas was higher than the control pasta only for the sample with 15% TP (Figure 2), while the linseed meal addition led to a decrease in carotenoids content, due to the dilution effect. On the other hand, in the pasta with linseed meal the tocols content increased, while in that with TP the rise was noticeable only after a 15% addition. These results were clearly linked to the characteristics of the raw materials (Table 2).

Supplementary Table S1 details the different carotenoids and tocols found in the pastas. As expected from the analysis of the raw materials, roughly 80% of the carotenoids in control and linseed meal enriched pastas was lutein. Notwithstanding the prevalence of lycopene + β-carotene in the tomato pomace, the pastas with this ingredient still had abundant lutein (about 50% of total carotenoids) because of the fava bean flour contribution; lycopene + β-carotene represented 39% and 43% of total carotenoids in the 10% and 15% enriched samples, respectively.

Padalino et al. [24] reported far superior levels of carotenoids: 51.6 and 133.6 mg/kg of β-carotene in 10% and 15% tomato-pomace-enriched pastas. However, about 40 mg/kg was reported for durum wheat control pasta, but this amount appears at least questionable cause it is one order of magnitude more than that reported by numerous authors for semolina [56,57,58,59,60], and pasta extrusion is further detrimental for carotenoids [59]. Kaur et al. [61] found 8.8 and 36.9 mg/kg carotenoids, primarily β-carotene, in 10% and 15% orange-pomace-enriched pastas, respectively. Likewise, 5.2 mg/kg DM, mainly lutein, are reported in 10% olive-pomace-enriched spaghetti [60].

The α- and β-tocopherols were found only in the TP enriched pastas: α-tocopherol, which exhibits the highest biological activity [62], was present in small amount (0.6–1.0 mg/kg DM), while 8.6 mg/kg DM were found in olive-pomace-enriched pasta [60]. The γ-tocopherol was prevalent in all pasta samples, ranging from 6.55 mg/kg DM (about 90% of total tocols) in the control to 10.27 mg/kg DM (about 80%) in pasta with 15% of TP. Conversely, the δ-tocopherol was present, in low concentrations, only in the TP-enriched samples and the β-tocotrienol was present (40%) in the linseed-meal-enriched pastas.

#### 2.2.3. Hydrophilic Antioxidants

**Figure 3:**
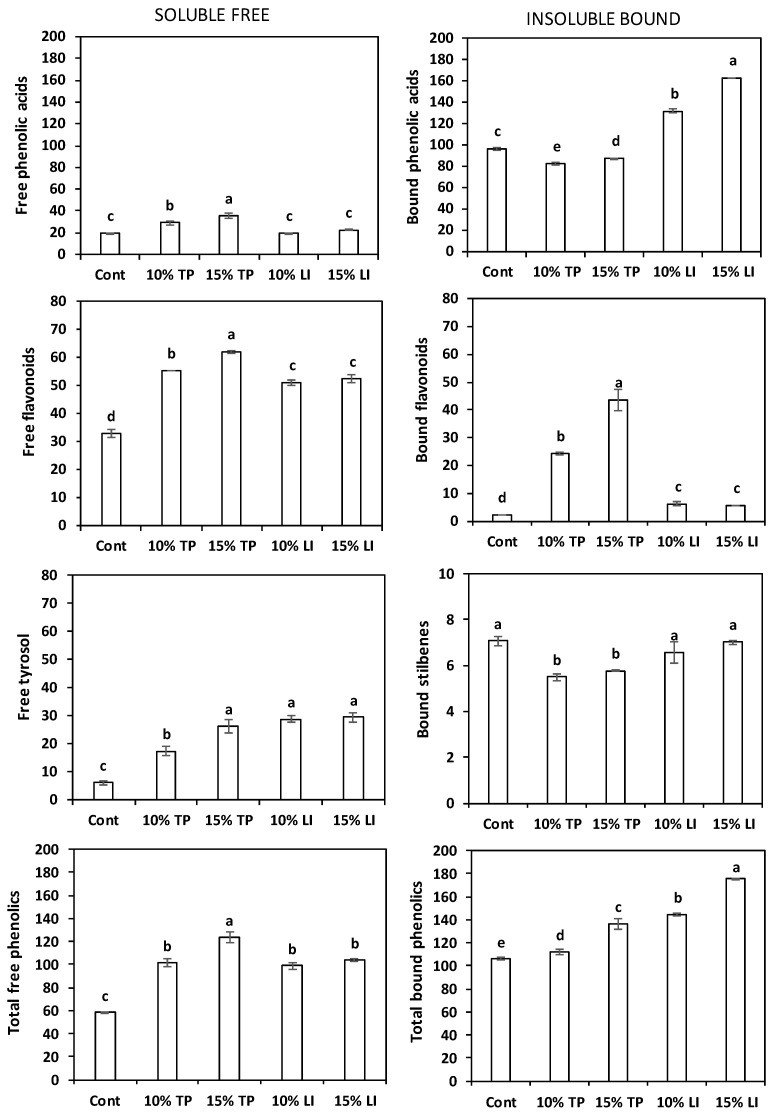
Total phenolic acids, total flavonoids, tyrosol, and total phenols content of the soluble free (**left**) and insoluble bound (**right**) fractions (mean ± standard deviation) of pasta prepared with rice flour and fava bean flour (Cont) or enriched with different percentages of tomato pomace (TP) or linseed meal (LI). The error bars represent the standard deviation; different letters indicate significant difference (*p* < 0.05) between the samples.

The total free polyphenol content of all the enriched pastas was significantly higher than that of the control pasta (Figure 3), in particular in the pasta with 15% of TP (123.42 mg/kg DM). The main polyphenols found in the enriched pastas (Supplementary Table S2) were catechin (36–49% of total free polyphenols), tyrosol (17–29%), and protocatechuic acid (16–19%). The total free phenolic acids were slightly lower than the value (34.02 mg/kg DM) reported by Oniszczuk et al. [63] in gluten free pasta made of rice and field bean (2/1; *w*/*w*) with a different phenolic profile; isoferulic acid was the main compound. Values of soluble phenolics like ours were found in egg pastas enriched with 7% pomace from grape or olive (91 and 96 mg/kg DM, respectively); their raw materials exhibited much higher concentration of phenols [64]. Similarly, 107.6 mg/kg DM of free phenolics were quantified in 10% olive-pomace-enriched pasta [60].

The total bound polyphenol content in enriched pastas (Figure 3) was significantly higher than in the control, especially in the one enriched with linseed meal. The bound flavonoids were more abundant in the pastas with tomato pomace (22–32%), while in those with linseed meal the bound phenolic acids represented >90% of the bound phenolics, as well as in the control. The individual polyphenols contents (Supplementary Table S2) reflected the composition of the flours: ferulic acid, present in rice flour, was plentiful in the control (86%) and in all enriched pastas (49–72% of total bound polyphenols), in particular in those with linseed meal; naringenin (11–16%) and quercetin (9–13%) were relevant in the pastas with tomato pomace, and protocatechuic acid (16–22%) in those with linseed meal. The total phenolics amount corresponded to 231–271 and 259–305 mg/kg DM for tomato- and linseed-enriched pastas, respectively, in accordance with the average value (245.08 mg/kg DM) reported by Padalino et al. [60].

Overall, the mass balance before and after pasta making showed a degradation of free carotenoids, tocols and polyphenols and a variable behaviour of the bound polyphenols.

#### 2.2.4. Antioxidant Capacity

**Figure 4:**
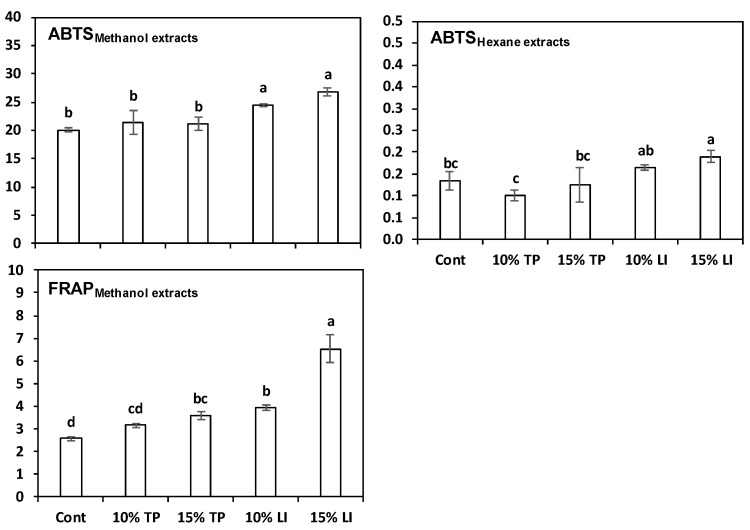
Antioxidant capacity (mean ± standard deviation) of extracts in 80% methanol (MeOH) or hexane of pasta prepared with rice flour and fava bean flour (Cont) or enriched with different percentages of tomato pomace (TP) or linseed meal (LI). The error bars represent the standard deviation; different letters indicate significant difference (*p* < 0.05) between the samples.

The pasta samples with linseed meal always showed the greatest antioxidant capacity (Figure 4). The pastas enriched with TP gave contrasting results: their methanolic extracts appeared similar to the control with ABTS method but superior with FRAP. The hexane extracts, instead, contained ABTS values inferior (TP 10%) or similar (TP 15%) to the control. These results largely agree with the raw materials findings because the antioxidant capacity of linseed meal was much higher than that of TP.

#### 2.2.5. Fracturability

**Figure 5:**
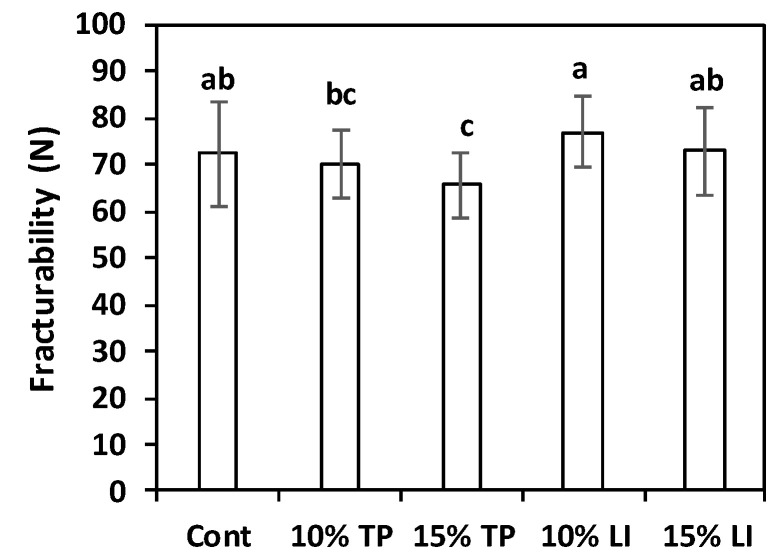
Fracturability of pasta prepared with rice flour and fava bean flour (Cont) or enriched with different percentages of tomato pomace (TP) or linseed meal (LI). The error bars represent the standard deviation; different letters indicate significant difference (*p* < 0.05) between the samples.

Raw pasta fracturability is an interesting textural parameter, useful to predict pasta behaviour during transport and storage: in fact, a high degree of mechanical strength can reduce breakage during handling. Tomato pomace and linseed meal addition to gluten-free pasta samples determined some changes in this parameter (Figure 5). The highest fracturability value was registered for the samples with 10% linseed meal (77.1 ± 7.7 N), while the pasta with 15% tomato pomace presented the lowest score (65.7 ± 6.9 N), significantly inferior to the control (72.4 ± 11.2 N); linseed 15% and tomato pomace 10% pasta samples showed intermediate results. Enriching bread wheat pasta with 5, 10, and 20% of spirulina reduced fracturability values [65]; similarly, a marked matrix weakening with ≥20% lupin flour incorporation was detected [66].

## 3. Materials and Methods

### 3.1. Materials

The control gluten-free pastas were prepared from a basic formulation of 66.7% rice flour and 33.3% fava bean flour. The rice flour (size inferior to 200 µm) was obtained from Bio Aglut Company (Constantine, Algeria) and the fava bean flour (size inferior to 200 µm) was obtained by grinding seeds purchased from Al-Amir Company (Housh Essa, Egypt). The enriched pastas were prepared by replacing 10% or 15% of the basic formulation with tomato waste (TP) obtained by grinding (after drying at 45 °C for 10 h) tomato pomace acquired from Zimba Canning Company (Guelma, Algeria), or with linseed meal (LI), a by-product of mechanical oil extraction by hydraulic press, supplied by Health Embassy LTD (Cheltenham, UK).

### 3.2. Methods

#### 3.2.1. Pasta Making

About 14 kg of the basic formulation and the water needed to reach 40% humidity were pre-treated with a PROGEL^®^ extruder (Braibanti, Milan, Italy), using the following parameters: extrusion screw temperature 130 °C; pellets outlet temperature 85–90 °C; pellets extrusion pressure 10 bar; length of pellets 5 mm. The pasta (macaroni format) was prepared in a Mac30 pilot plant (Italpast, Parma, Italy) equipped with a pre-kneading tank, a vacuum kneading tank, an extrusion cylinder thermostated with water at 20 °C and a Teflon die. About 3.0 kg of basic formulation (control), or of mixtures enriched with TP or LI, and the water needed to reach 40% humidity were mixed for 12–15 min, then the mix was extruded under vacuum (−0.9 bar) at a pressure of 65–70 bar. A jacket with cold water maintained the temperature of the dough at 40 °C. The pasta samples were dried in an experimental cell (Braibanti, Milan, Italy) using a long drying cycle (17 h) at low temperature (max 60 °C) and relative humidity of 75%. All the dry pasta samples were stored at room temperature until analysis.

Just before the chemical analyses, the samples were ground at 18,000 rpm for 20 s with a Waring Heavy Duty Blender mill (Waring Commercial, Torrington, CT, USA) using a special blade for dusty materials, and then sieved through a n° 18 steel mesh to exclude particles greater than 1 mm. The ground samples were stored in sealed vacuum bags at −20 °C until analysis.

#### 3.2.2. Chemical Composition of the Raw Materials and Pasta Samples

The humidity of the raw materials was determined gravimetrically according to AACC method 44–15.02 [67]. The protein content (g/100 g DM) of raw materials and pastas was assessed following the Kjeldahl approach as described in AOAC method 979.09 [68], using the conversion factor 6.25. Total lipid content of the tomato by-product and linseed meal was determined by the gravimetric method after Soxhlet extraction using petroleum ether as a solvent (Ba 3–38 method; AOCS) [69]; for rice and fava bean flours and pasta, the Soxhlet extraction was carried out after acid hydrolysis of the samples, following ICC method 136 [70]. The determination of the ash of raw materials and pastas was carried out according to AOAC method 923.03 [68] by incineration at 600 °C using a Tactical 308 muffle (Gallenkamp, Cambridge, UK). The crude fibre of the pastas was determined with the FT 122 Fibertec™ apparatus (Foss, Hillerød, Denmark) according to the Weende technique as reported in AOAC method 978.10 [68], based on the solubilization of the non-cellulosic compounds by treatment with a boiling solution of 0.255 N sulfuric acid and subsequently with a boiling solution of 0.313 N sodium hydroxide.

Furosine, an index of heat damage in several food products, was assessed in the pastas by HPLC as described by Hidalgo and Brandolini [71].

#### 3.2.3. Tocols, Carotenoids, and Phenols Quantification

Tocopherols and carotenoids extracts from raw materials and pastas were obtained by saponification [72]. Briefly: 2 g samples were exactly weighted in a screw-capped tube and saponified under nitrogen for 45 min at 70 °C, with the addition of 5 mL ethanolic pyrogallol (60 g/L) as antioxidant, 2 mL ethanol (95%), 2 mL sodium chloride (10 g/L), and 2 mL potassium hydroxide (600 g/L). Afterwards, they were cooled in an ice bath and 15 mL sodium chloride (10 g/L) was added. The suspension was then extracted twice with 15 mL hexane:ethyl acetate (9:1 *v*/*v*). The organic layer was collected and evaporated under vacuum, followed by nitrogen drying; the residue was dissolved in 2 mL hexane:isopropyl alcohol (99:1 *v*/*v*) and filtered through a 0.22 mm PTFE membrane.

Tocols [73] and carotenoids [74] quantification was carried out by normal-phase HPLC. The operating conditions for tocol quantification were: Adamas^®^ Silica column 250 × 4.6 mm, 5 μm, and guard cartridge 10 × 4.6 mm, 5 μm (Sepachrom SRL, Rho, Italy); mobile phase—hexane:ethyl acetate:acetic acid (97.3:1.8:0.9, *v*/*v*/*v*); flow rate—1.6 mL/min; pump L-2130 Elite LaChrom (Hitachi, Tokyo, Japan); fluorimetric detector—Jasco 821 FP Intelligent Spectrofluorometer (Tokyo, Japan) at excitation–emission wavelengths of 290 nm and 330 nm, respectively; and connected to a computer with the software Empower 2 (Waters Chromatography Division, Millipore, Milford, MA, USA) through the Waters e-SAT/IN module

The operating conditions for carotenoid detection were: Adamas^®^ Silica column 250 × 4.6 mm, 5 μm, and guard cartridge 10 × 4.6 mm, 5 μm (Sepachrom SRL, Rho, Italy); column oven at 20 °C L-2300 Elite LaChrom (Hitachi, Tokyo, Japan); mobile phase—hexane:isopropyl alcohol (5%); flow rate—1.5 mL/min; pump L-2130 Elite LaChrom (Hitachi, Tokyo, Japan). Carotenoids were detected at 445 nm by Diode Array Detector L2450 Elite LaChrom (Hitachi, Tokyo, Japan) set in the range of 200–650 nm. The HPLC system was controlled by the software EZChrom Client/Server version 3.1.7. All the compounds were quantified by the external standard method. Chromatograms of each group of compound are shown in Supplementary Figure S1.

Soluble free and insoluble bound phenolics of raw materials and pastas, extracted as described by Nakov et al. [75] and by Yilmaz et al. [76], respectively, were analysed by reverse-phase HPLC [77] using an HPLC system with an Adamas^®^ C18-AQ 5 μm 4.60 mm × 250 mm column and a C18 5 μm 4.60 mm × 10.0 mm precolumn (Sepachrom srl, Rho, Italy), thermostated at 30.0 °C; L-2130 pump, L-2300 column oven and L2450 Diode Array Detector Elite LaChrom (Hitachi, Tokyo, Japan). All the compounds were quantified by the external standard method. Chromatograms of free and bound phenolics are shown in Supplementary Figure S2.

#### 3.2.4. Antioxidant Capacity

In pastas, the antioxidant capacity of the lipophilic compounds was determined by the ABTS method [78] after hexane extraction, while that of the hydrophilic compounds was assessed by the ABTS [78] and FRAP [17] methods after methanol:water (80:20 *v*:*v*) extraction. Exactly 0.200 ± 0.010 g of ground pasta were resuspended with 1 mL of 80% methanol solution or 1 mL hexane in an Eppendorf tube, vortexed, placed in an ultrasonic bath for 10 min, shaken for 20 min, and centrifuged at 12,000 rpm for 10 min. After recovering the supernatant, the extraction was repeated, and the two supernatants combined. The tests were performed on three independent samples and the results are expressed as mmol Trolox equivalent (TE)/kg.

All the chemical analyses were performed on three independent samples.

#### 3.2.5. Pasta Fracturability

Dry pasta fracturability was assessed using a Texture Analyser (TA.XT PLUS, Stable Micro Systems, Surrey, UK) equipped with a 5 kg load cell and Software EXPONENT. Pasta sample was compressed at a test speed of 2 mm/s with a rectangular probe (30 mm × 50 mm) set at a width of 12 mm. Fracturability was measured as the maximum force needed to break the pasta. The average value of 15 replicates is reported.

#### 3.2.6. Statistical Analysis

To evaluate the effect of the different flours and pasta formulations, the data were processed by one-way analysis of variance (ANOVA), considering the type of mixture as a factor. Before the ANOVAs, the data normal distribution was verified and, if necessary, logarithmic transformations were performed. When significant differences were found (*p* < 0.05), Fisher’s least significant difference (LSD) at 95% significance was computed. All analyses were performed using the statistical program STATGRAPHICS^®^ Centurion (Statpoint Technologies, Inc., Warrenton, VA, USA). The means and standard deviations were calculated using the Excel program (Microsoft^®^, Redmond, WA, USA).

## 4. Conclusions

The enriched pastas were richer in fibre and lipids than the control, while protein and ash values were similar; the heat damage, assessed by furosine content, was always limited. The addition of tomato pomace or linseed meal improved the content of tocols but not the content of carotenoids. The free soluble polyphenols increase was similar for both by-products and proportional to the enrichment percentage, while the bound insoluble polyphenols were higher in the pastas with linseed meal. The antioxidant capacity of the free methanolic fraction was greater than that of the hexane extract. In conclusion, the addition of tomato and linseed by-products significantly increased the presence of bioactive compounds, particularly of polyphenols, in the enriched pastas. Future studies will be necessary to assess the stability and bioavailability of these health-improving molecules during cooking and digestion.

## Figures and Tables

**Table 1:** Content in protein, ash, lipid, and furosine (mean ± standard deviation) of rice flour, fava bean flour, tomato waste, and linseed meal.

	Rice	Fava Bean	Tomato By-Product	Linseed Meal
Moisture (g/100 g)	11.38 ± 0.17 ^a^	11.60 ± 0.55 ^a^	8.77 ± 0.20 ^b^	8.28 ± 0.61 ^b^
Protein (g/100 g DM)	7.57 ± 0.31 ^c^	26.78 ± 0.74 ^a^	14.92 ± 0.56 ^b^	26.69 ± 1.15 ^a^
Ash (g/100 g DM)	0.22 ± 0.01 ^d^	3.24 ± 0.00 ^c^	4.43 ± 0.02 ^b^	5.54 ± 0.00 ^a^
Lipid (g/100 g DM)	0.76 ± 0.01 ^d^	2.08 ± 0.03 ^c^	5.48 ± 0.17 ^b^	16.92 ± 0.09 ^a^
Furosine (mg/100 g protein)	nd ^d^	7.45 ± 0.51 ^b^	12.61 ± 0.17 ^a^	3.85 ± 0.65 ^c^

nd, lower than the detection limit. Different letters indicate significant difference (*p* < 0.05) between samples in the row.

**Table 2:** Content in carotenoids and tocols (mean ± standard deviation mg/kg DM) of rice flour, fava bean flour, tomato waste, and linseed meal.

	Rice	Fava Bean	Tomato By-Product	Linseed Meal
*Carotenoids*				
Lycopene + β-carotene	0.06 ± 0.01 ^c^	0.32 ± 0.11 ^b^	8.72 ± 1.35 ^a^	0.48 ± 0.26 ^b^
β-cryptoxanthin	nd	0.02 ± 0.01	0.09 ± 0.01	0.08 ± 0.02
Lutein	nd ^c^	5.26 ± 0.28 ^a^	1.66 ± 0.12 ^b^	1.72 ± 0.24 ^b^
Zeaxanthin	0.02 ± 0.01 ^b^	0.12 ± 0.04 ^b^	0.33 ± 0.09 ^a^	0.14 ± 0.06 ^b^
Total	0.08 ± 0.00 ^c^	5.73 ± 0.44 ^b^	10.79 ± 1.57 ^a^	2.43 ± 0.58 ^c^
*Tocols*				
α-tocopherol	nd ^c^	5.93 ± 0.14 ^b^	17.23 ± 1.23 ^a^	nd ^c^
α-tocotrienol	nd ^b^	nd ^b^	nd ^b^	2.19 ± 0.06 ^a^
β-tocopherol	nd	1.05 ± 0.67	1.40 ± 0.12	1.56 ± 0.08
β-tocotrienol	nd ^b^	nd ^b^	nd ^b^	106.38 ± 1.73 ^a^
γ-tocopherol	nd ^d^	82.97 ± 0.38 ^a^	68.58 ± 4.82 ^b^	48.66 ± 0.28 ^c^
γ-tocotrienol	0.89 ± 0.02 ^a^	0.71 ± 0.09 ^b^	nd ^c^	nd ^c^
δ-tocopherol	nd ^c^	0.99 ± 0.17 ^a^	0.92 ± 0.09 ^a^	0.50 ± 0.02 ^b^
δ-tocotrienol	nd ^b^	nd ^b^	3.60 ± 0.28 ^a^	nd ^b^
Total	0.89 ± 0.02 ^c^	91.65 ± 0.17 ^b^	91.72 ± 6.55 ^b^	159.28 ± 2.16 ^a^

nd, lower than the detection limit. Different letters indicate significant difference (*p* < 0.05) between samples in the row.

**Table 3:** Composition (mean ± standard deviation; mg/kg DM) of soluble free and insoluble bound polyphenols in rice flour, fava bean flour, tomato waste, and linseed meal.

	Rice	Fava Bean	Tomato By-Product	Linseed Meal
*Free phenolic acids*				
Protocatechuic	nd ^c^	305.07 ± 0.49 ^a^	37.31 ± 1.31 ^b^	na
4-hydroxybenzoic	nd ^c^	8.14 ± 1.06 ^b^	24.38 ± 0.66 ^a^	na
Syringic	nd ^c^	72.14 ± 2.72 ^b^	108.80 ± 7.54 ^a^	na
*p*-coumaric	nd ^c^	0.14 ± 0.01 ^b^	1.81 ± 0.00 ^a^	na
Ferulic	nd ^c^	1.36 ± 0.05 ^b^	15.79 ± 0.43 ^a^	na
Total	nd ^c^	386.85 ± 2.22 ^a^	188.10 ± 5.14 ^b^	na
*Free flavonoids*				
Catechin	nd ^b^	659.13 ± 38.5 ^a^	nd ^b^	na
Epicatechin	nd ^c^	40.37 ± 1.81 ^b^	52.26 ± 1.49 ^a^	na
Rutin	nd ^b^	nd ^b^	133.83 ± 2.64 ^a^	na
Quercetin	nd ^b^	nd ^b^	15.11 ± 0.02 ^a^	na
Quercetin derivative	nd ^c^	3.97 ± 0.08 ^b^	70.21 ± 2.20 ^a^	na
Naringenin	nd ^b^	nd ^b^	125.05 ± 1.27 ^a^	na
Apigenin	nd ^b^	nd ^b^	6.74 ± 0.17 ^a^	na
Total	nd ^c^	703.46 ± 40.25 ^a^	403.20 ± 0.85 ^b^	na
*Phenylethanoids*				
Tyrosol	nd ^c^	47.45 ± 3.13 ^b^	455.44 ± 10.01 ^a^	na
Total free phenolics	nd ^b^	1137.76 ± 45.84 ^a^	1046.74 ± 4.02 ^a^	na
*Bound phenolic acids*				
Protocatechuic	2.16 ± 0.08 ^c^	0.25 ± 0.00 ^c^	13.95 ± 0.75 ^b^	180.10 ± 5.35 ^a^
4-hydroxybenzoic	1.08 ± 0.14 ^c^	nd ^d^	22.03 ± 0.26 ^a^	7.56 ± 0.13 ^b^
Caffeic	0.14 ± 0.02 ^c^	nd ^d^	14.51 ± 10.40 ^a^	1.33 ± 0.09 ^b^
*p*-coumaric	0.19 ± 0.10 ^c^	0.06 ± 0.00 ^c^	9.49 ± 0.53 ^a^	2.71 ± 0.02 ^b^
Sinapic	0.31 ± 0.02 ^b^	0.65 ± 0.29 ^b^	nd	2.49 ± 0.01 ^a^
Ferulic	100.46 ± 7.56 ^b^	4.54 ± 0.08 ^d^	32.60 ± 0.40 ^c^	144.16 ± 3.98 ^a^
Cinnamic derivative	0.66 ± 0.02 ^c^	0.63 ± 0.01 ^c^	40.24 ± 1.03 ^a^	1.35 ± 0.04 ^b^
Total	105.00 ± 7.59 ^c^	6.13 ± 0.37 ^d^	132.80 ± 11.35 ^b^	339.69 ± 9.53 ^a^
*Bound flavonoids*				
Epicatechin	nd ^c^	nd ^c^	121.38 ± 2.63 ^a^	8.97 ± 0.56 ^b^
Quercetin	0.79 ± 0.33 ^c^	nd ^d^	201.31 ± 5.91 ^a^	2.50 ± 0.04 ^b^
Naringenin	0.30 ± 0.09 ^c^	nd ^d^	590.47 ± 2.18 ^a^	3.82 ± 0.13 ^b^
Total	1.10 ± 0.24 ^c^	nd ^d^	913.16 ± 6.37 ^a^	15.29 ± 0.39 ^b^
*Bound stilbenes*				
Resveratrol derivative	2.13 ± 0.41 ^a^	0.55 ± 0.02 ^b^	nd ^c^	1.44 ± 0.02 ^a^
Resveratrol	4.54 ± 0.74 ^b^	0.67 ± 0.04 ^c^	nd ^d^	7.80 ± 1.21 ^a^
Total	6.67 ± 1.15 ^b^	1.22 ± 0.0 ^c^	nd ^d^	9.24 ± 1.19 ^a^
Total bound phenolics	112.77 ± 8.49 ^c^	7.35 ± 0.35 ^d^	1046.0 ± 17.7 ^a^	364.22 ± 8.74 ^b^

nd, lower than the detection limit; na, not available. Different letters indicate significant difference (*p* < 0.05) between samples in the row.

## Data Availability

Not applicable.

## Supplementary Materials

The following supporting information can be downloaded at: https://www.mdpi.com/article/10.3390/molecules27248993/s1, Figure S1: HPLC chromatograms of carotenoids and tocols in the pasta sample enriched with 15% of tomato pomace. Figure S2: HPLC chromatograms at 280 nm of the free and bound phenolics in the pasta sample enriched with 15% of tomato pomace. Caffeic, sinapic, and resveratrol are read at 320 nm, while rutin and quercetin at 360 nm. Table S1: Composition of carotenoids and tocols (mean ± standard deviation; mg/kg DM) in pasta prepared with rice and fava bean flours (Control) or enriched with different percentages of tomato waste (TP) or linseed meal (LI). Table S2: Composition (mean ± standard deviation) of soluble-free and insoluble-bound polyphenols; mg/kg DM) in pasta prepared with rice and fava bean flours (Control) or enriched with different percentages of tomato waste (TP) or linseed meal (LI).
