# Supplementary material for: Antioxidant Properties of Gluten-Free Pasta Enriched with Vegetable By-Products
Source: Molecules. 2022 Dec 16;27(24):8993. doi: 10.3390/molecules27248993 (PMC9784952; doi:10.3390/molecules27248993)
Supplement: Supplementary file 1 [file molecules-27-08993-s001.zip › molecules-2046758-supplementary.pdf]

Table S1. Composition of carotenoids and tocols (mean ± standard deviation; mg/kg DM) in pasta prepared with rice and faba bean flours (Control) or enriched with different percentages of tomato waste (TP) or linseed meal (LI).

	Control	10% TP	15% TP	10% LI	15% LI
Carotenoids					
Lycopene + β-carotene	$0.09^{c} \pm 0.03$	$0.48^{b} \pm 0.01$	$0.61^{a} \pm 0.03$	$0.06^{c} \pm 0.01$	$0.06^{c} \pm 0.00$
β-cryptoxanthin	0.06 ± 0.01	0.05 ± 0.00	0.06 ± 0.01	0.05 ± 0.01	0.05 ± 0.00
Lutein	$0.93^{a} \pm 0.01$	$0.65^{b} \pm 0.03$	$0.68^{b} \pm 0.02$	$0.80^{ab} \pm 0.12$	$0.76^{b} \pm 0.03$
Zeaxanthin	0.09 ± 0.00	0.04 ± 0.01	0.06 ± 0.01	0.12 ± 0.11	0.04 ± 0.00
Tocols					
α-tocopherol	nd	0.34 ± 0.32	0.94 ± 0.02	nd	nd
β-tocopherol	nd	0.39 ± 0.25	0.50 ± 0.05	nd	nd
β-tocotrienol	nd	nd	nd	$4.93^{b} \pm 0.25$	$8.35^{a} \pm 0.47$
γ-tocopherol	$6.55^{c} \pm 0.20$	$7.22^{c} \pm 0.59$	$10.27^{a} \pm 0.47$	$7.40^{c} \pm 0.23$	$8.81^{b} \pm 0.61$
γ-tocotrienol	0.85 ± 0.01	0.70 ± 0.14	0.55 ± 0.14	0.82 ± 0.04	0.70 ± 0.11
δ-tocotrienol	nd	0.48 ± 0.12	0.55 ± 0.08	nd	nd

nd, lower than the detection limit.

Table S2. Composition (mean ± standard deviation) of soluble free and insoluble bound polyphenols; mg/kg DM) in pasta prepared with rice and fava bean flours (Control) or enriched with different percentages of tomato waste (TP) or linseed meal (LI).

	Control	10% TP	15% TP	10% LI	15% LI
**Soluble free phenolics**					
Phenolic acids					
Protocatechuic	15.94 ± 0.89	19.35 ± 2.03	19.36 ± 1.97	15.52 ± 0.28	18.69 ± 0.57
4-hydroxybenzoic	0.54^{c} ± 0.03	1.21^{b} ± 0.08	1.80^{a} ± 0.31	1.58^{ab} ± 0.38	1.19^{b} ± 0.03
Syringic	1.77^{d} ± 0.33	6.65^{b} ± 0.33	11.73^{a} ± 0.13	2.17^{cd} ± 0.10	2.48^{c} ± 0.07
p-coumaric	0.16 ± 0.01	0.15 ± 0.05	0.21 ± 0.09	nd	nd
Ferulic	0.96^{b} ± 0.15	1.69^{a} ± 0.09	2.19^{a} ± 0.23	nd	nd
Flavonoids					
Catechin	30.5^{c} ± 1.1	45.7^{b} ± 0.1	44.7^{b} ± 0.2	48.9^{a} ± 0.8	49.8^{a} ± 1.7
Epicatechin	2.28 ± 0.24	1.90 ± 0.10	1.78 ± 0.36	2.05 ± 0.28	2.44 ± 0.20
Rutin	nd	0.66^{b} ± 0.04	3.74^{a} ± 0.44	nd	nd
Quercetin	nd	1.08^{b} ± 0.02	1.40^{a} ± 0.04	nd	nd
Naringenin	nd	5.83^{b} ± 0.12	10.46^{a} ± 0.48	nd	nd
Tyrosol	5.96^{c} ± 0.77	17.24^{b} ± 1.68	26.05^{a} ± 2.36	28.58^{a} ± 1.16	29.23^{a} ± 1.75
**Insoluble bound**					
Phenolic acids					
Protocatechuic	1.78^{c} ± 0.31	2.38^{c} ± 0.54	2.65^{c} ± 0.38	23.26^{b} ± 0.41	38.04^{a} ± 0.06
4-hydroxybenzoic	1.86^{c} ± 0.08	5.16^{a} ± 0.91	6.34^{a} ± 0.10	2.95^{bc} ± 1.56	4.41^{ab} ± 0.12
Caffeic	0.17^{b} ± 0.14	0.87^{b} ± 0.08	2.20^{a} ± 0.88	0.65^{b} ± 0.31	1.08^{b} ± 0.02
p-coumaric	0.57^{d} ± 0.06	1.11^{bc} ± 0.02	1.67^{a} ± 0.16	0.87^{cd} ± 0.43	1.47^{ab} ± 0.00
Sinapic	0.06^{c} ± 0.04	0.28^{bc} ± 0.12	0.42^{bc} ± 0.18	0.79^{b} ± 0.56	1.92^{a} ± 0.02
Ferulic	91.4^{c} ± 1.7	67.7^{d} ± 1.3	67.1^{d} ± 0.5	102.3^{b} ± 1.4	115.4^{a} ± 0.3
Cinnamic derivative	0.62^{c} ± 0.04	4.69^{b} ± 0.34	6.89^{a} ± 0.36	0.94^{c} ± 0.03	0.67^{c} ± 0.04
Flavonoids					
Epicatechin	1.07^{c} ± 0.01	1.89^{b} ± 0.63	4.16^{a} ± 0.29	2.45^{b} ± 0.09	4.74^{a} ± 0.13
Quercetin	1.05^{c} ± 0.17	9.87^{b} ± 0.78	17.12^{a} ± 2.00	1.77^{c} ± 0.08	0.58^{c} ± 0.05
Naringenin	0.10^{c} ± 0.09	12.53^{b} ± 1.07	22.32^{a} ± 2.17	2.01^{c} ± 0.70	0.34^{c} ± 0.04
Stilbenes					
Resveratrol derivative	2.80 ± 0.06	2.18 ± 0.02	2.35 ± 0.01	2.35 ± 0.50	2.41 ± 0.00
Resveratrol	4.27 ± 0.25	3.31 ± 0.13	3.43 ± 0.03	4.23 ± 0.27	4.62 ± 0.08

nd, lower than the detection limit.

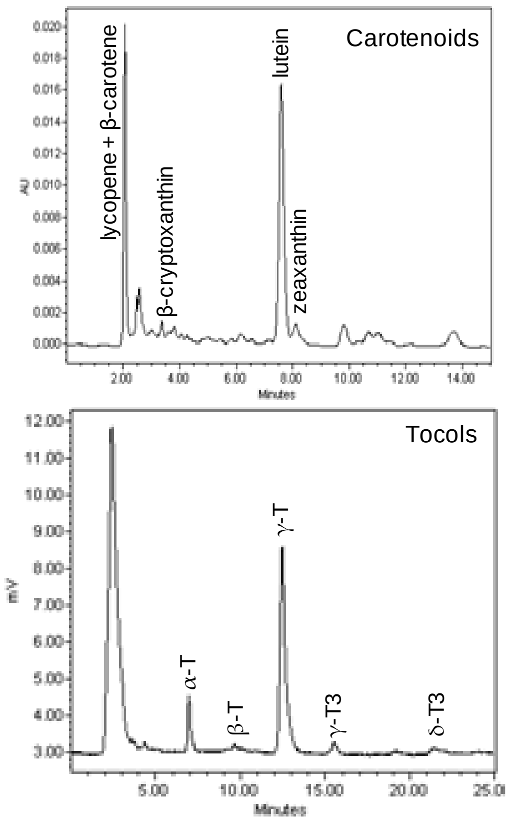

Figure S1. HPLC chromatograms of carotenoids and tocols in the pasta sample enriched with 15% of tomato pomace. T, tocopherol; T3, tocotrienol.

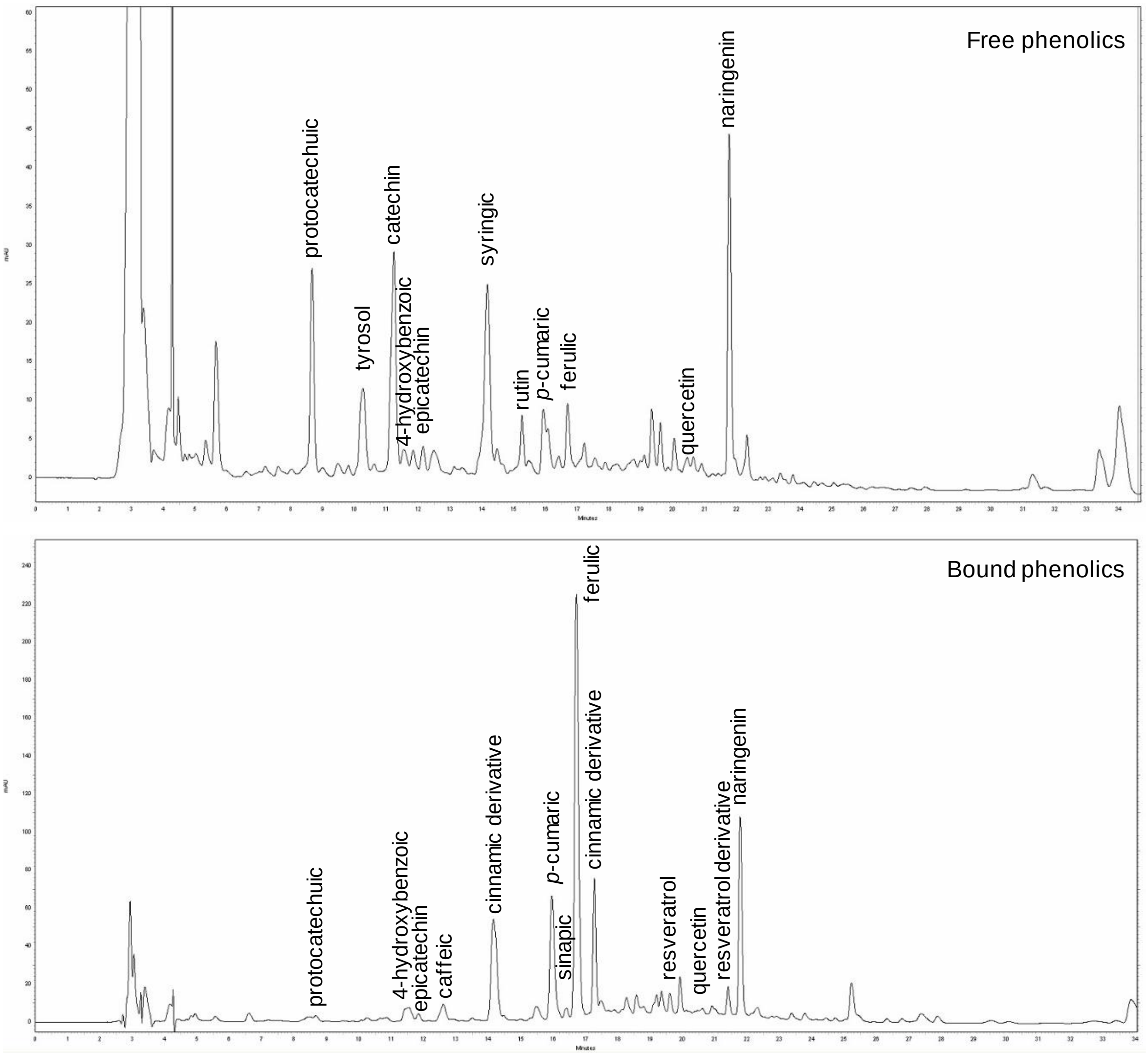

Figure S2. HPLC chromatograms at 280 nm of the free and bound phenolics in the pasta sample enriched with 15% of tomato pomace. Caffeic, sinapic and resveratrol are read at 320 nm while rutin and quercetin at 360 nm.
